# Microbiota-based therapies in oral health and disorders

**DOI:** 10.1007/s12223-025-01324-x

**Published:** 2025-10-11

**Authors:** Akram N. Salah, Youssef A. Doghish, Shaimaa O. Abbass, Reda M. Mansour, Ghadir A. Sayed, Nourhan H. Elshami, Sherif S. Abdel Mageed, Osama A. Mohammed, Ahmed I. Abulsoud, Mohamed Bakr Zaki, Esraa M. Mosalam, Mahmoud A. Elrebehy, Kareem Alfarsi, Ahmed S. Doghish

**Affiliations:** 1 Microbiology and Immunology Department, Faculty of Pharmacy, Badr University in Cairo (BUC), Badr City, 11829 Cairo Egypt; 2 Faculty of Dentistry, Badr University in Cairo (BUC), Badr City, 11829 Cairo Egypt; 3 Faculty of Dentistry, Ain Shams University, Cairo, 11566 Egypt; 4 Zoology and Entomology Department, Faculty of Science, Helwan University, Helwan, Egypt; 5 Molecular Biology and Biotechnology Department, School of Biotechnology, Badr University in Cairo (BUC), Badr City, 11829 Cairo Egypt; 6 Department of Biochemistry, Faculty of Pharmacy, Egyptian Russian University, Cairo, Egypt; 7 Department of Microbiology and Immunology, Faculty of Pharmacy, Organization of African Unity St., Ain Shams University, POB: 11566, Abbassia, Cairo Egypt; 8 Pharmacology and Toxicology Department, Faculty of Pharmacy, Badr University in Cairo (BUC), Badr City, 11829 Cairo Egypt; 9 Department of Pharmacology, College of Medicine, University of Bisha, 61922 Bisha, Saudi Arabia; 10 Faculty of Pharmacy, Integrative Health Centre, Heliopolis University, Cairo, 11785 Egypt; 11 Biochemistry and Molecular Biology Department, Faculty of Pharmacy (Boys), Al-Azhar University, Nasr City, 11231 Cairo Egypt; 12 Department of Biochemistry, Faculty of Pharmacy, University of Sadat City, Sadat City, 32897 Menoufia Egypt; 13 Department of Biochemistry, Faculty of Pharmacy, Menoufia National University, Km Cairo-Alexandria Agricultural Road, Menofia, Egypt; 14 Biochemistry Department, Faculty of Pharmacy, Menoufia University, Shebin El-Kom, 32511 Egypt; 15 Department of Pharm D, Faculty of Pharmacy, Jadara University, Irbid, 21110 Jordan; 16 Department of Biochemistry, Faculty of Pharmacy, Galala University, New Galala CitySuez, 43713 Egypt; 17 Surgical Intensive Care Unit at 57357 Children’s Cancer Hospital, Cairo, Egypt; 18 Department of Biochemistry, Faculty of Pharmacy, Badr University in Cairo (BUC), Badr City , 11829 Cairo Egypt

**Keywords:** Oral microbiome, Oral diseases, Microbiota therapeutics, Dental caries, Periodontal disease, Probiotics

## Abstract

The human oral microbiome is a complex, dynamic ecosystem critically involved in maintaining oral health and contributing to systemic well-being. Many bacteria and fungi are involved in oral cavities such as *Penicillium*, *Rhodotorula*, *Saccharomycetales*,* Streptococcus*, *Veillonella*,* Neisseria*, *Actinomyces*, and *Schizophyllum.* Disruption of microbial homeostasis, or dysbiosis, underpins a wide spectrum of oral diseases, including dental caries, periodontal disease, endodontic infections, and mucosal conditions. Recent advances in microbiome research have elucidated the mechanisms by which pathogenic microbial consortia, such as the red complex (*Porphyromonas gingivalis*, *Tannerella. forsythia*, and* Treponema denticola*), synergistically promote disease progression through virulence factors, metabolic interactions, and biofilm formation. Emerging microbiome-based therapies, comprising probiotics, postbiotics, predatory bacteria, and using bacteriophages, offer promising adjuncts or alternatives to traditional antimicrobial approaches by restoring microbial balance, reducing pathogenic load, and modulating host immune responses. For instance, probiotic strains like* Streptococcus salivarius* and *Lactobacillus* spp. have demonstrated efficacy in reducing plaque, gingival inflammation, and pathogenic bacteria, as well as having significant immunological modulation, while postbiotics provide similar benefits with enhanced safety and stability. Additionally, predatory bacteria such as* Bdellovibrio bacteriovorus *show potential for selective bacterial elimination and combating periodontal diseases that are driven by Gram-negative anaerobes. Bacteriophages offer another precision tool for targeting oral pathogens by lysing bacteria upon replication. Finally, oral microbiota transplantation aimed at treating periodontal disease by restoring a balanced microbial community in the oral cavity. These innovative strategies, combined with a nuanced understanding of biofilm dynamics and host–microbe interactions, pave the way for personalized and ecologically sustainable oral health interventions. Continued research is essential to translate these promising approaches into clinical practice, optimize delivery systems, and elucidate long-term safety and efficacy.

## Introduction

The oral cavity serves as the nexus of medicine and dentistry, providing insight into a person’s overall health (Maier 2023). Oral health refers to the condition of the teeth, mouth, and orofacial structures that allow individuals to carry out vital functions such as breathing, eating, and speaking, while also incorporating psychosocial aspects like self-esteem, well-being, and the capacity to socialize and work without discomfort, pain, or embarrassment. Oral health fluctuates over the lifespan, from infancy to old age, and is essential to overall health, enabling individuals to engage in society and realize their potential (Woo et al. 2022; Abdelaziz et al. 2025).

Oral disorders rank among the most prevalent afflictions in humans. These disorders constitute significant public health concerns globally, affecting individuals and communities through pain, functional limitations, and diminished quality of life (Warnakulasuriya et al. 2021). The prevalence of oral diseases is greatest among marginalized and impoverished communities, affected by their living situations, behaviors, and environmental influences. Enhancing public health initiatives and executing preventive strategies are essential for tackling oral health issues worldwide (Kambara et al. 2025).

Oral diseases, despite their significant social and economic consequences, receive less attention in numerous nations and remain an overlooked aspect of global health. They can cause significant functional limits, discomfort, and pain, resulting in disability (social or psychological), impairment, and handicap (Sakanaka 2024). Numerous diseases have been documented in the literature, including dental caries, periodontal diseases, various oral infections, oral precancerous lesions, oral cancer, salivary gland disorders, temporomandibular joint (TMJ) disorders, and various systemic disorders associated with oral manifestations (Shinde et al. 2022).

The microbiome constitutes an aspect of the environment that accompanies individuals. It encompasses all microorganisms, including fungi, bacteria, and viruses, together with their genetic material, residing in a certain habitat, such as the human body (de Vos et al. 2022). It is a dynamic community of microorganisms that alters in response to many circumstances, such as exercise, nutrition, and medicine (Joos 2025). The microbiome is essential for human health, influencing digestion, food fermentation, immune response stimulation, pathogen defense, vitamin synthesis, immunity, and mental health (Lin et al. 2022).

The oral microbiota is the second most significant and the most diverse microbial community, following the gut (Li et al. 2022a). The Human Oral Microbiome Database has over 1100 distinct taxonomic groupings, including the genera *Streptococcus*, *Veillonella*, *Neisseria*, and *Actinomyces*, linked to the core microbiome (Baker et al. 2017).

Previous studies tried to find the association between the oral microbiome and other diseases. Scher et al. reported that the subgingival microbiota, specifically, *Anaeroglobus geminatus*, was significantly correlated with some oral and autoimmune diseases (Scher et al. 2012). As well, Santacroce et al. found that there is an increased presence of oral microbiota at the first stage of pregnancy, such as *Aggregatibacter actinomycetemcomitans* and *Porphyromonas gingivalis* in the gingival sulcus, whereas *Candida* becomes more prevalent during the final trimester, alongside *Capnocytophaga sputigena*,* Fusobacterium nucleatum naviforme*, *Prevotella melaninogenica*,* Streptococcus sanguinis*, *Veillonella parvula*, *Selenomonas noxia*, and other microorganisms that are also found in the oral cavity which play a great role in human body ecosystem (Santacroce et al. 2023). Additionally, Nascimento reported that caries and periodontitis are intricately linked to a dysbiosis of microbial consortia induced by environmental alterations, leading to the proliferation of acidogenic and acidophilic organisms in supragingival biofilms. This selective process disrupts the pH homeostasis of biofilms and shifts the demineralization-remineralization balance toward the depletion of tooth (Nascimento 2017).

There are many challenges attributed to the management of oral disorders that encompass ongoing gaps in access to care, the increasing incidence of lifestyle-related diseases, and the rise of antibiotic resistance. Addressing these necessitates a multifaceted strategy, encompassing the promotion of oral health literacy, enhancement of access to preventive treatments, and the development of alternative antimicrobial methods relating to microbiome-based therapeutic approaches (Spatafora et al. 2024).

Microbiome-based therapies, which involve the manipulation of microbiota, are emerging as promising strategies to address microbial dysbiosis and the consequent dysregulation of the immune system (Shinde et al. 2022; Gulliver 2022). Contemporary therapeutic approaches, including probiotics, fecal microbiota transplantation (FMT), and interventions utilizing microbial metabolites, have demonstrated disparate levels of efficacy (Renvert et al. 2020). Nonetheless, their practical implementation is frequently obstructed by elements including substantial inter-individual variability of microbiomes, the intricacy of microbial interactions, and considerable deficiencies in mechanistic comprehension of the molecular functioning of these medicines (Manrique et al. 2024; Rajasekaran et al. 2024).

Additionally, regulatory obstacles and the absence of established protocols further hinder their extensive clinical use. These limitations highlight the pressing necessity for advanced research to elucidate the fundamental mechanisms of host–microbiome interactions and to enhance microbiome-based therapies for more reliable and predictable clinical results (Poulose et al. 2024; Yaqub et al. 2025). Therefore, this study aims to elucidate the main novel microbiota-based therapies in oral disorders and illustrate their role in maintaining oral health among the population (Homayouni Rad et al. 2023).

Chronic or recurrent psychological stress induces dental and orthodontic disorders through inflammatory mechanisms and oxidative damage. Age-associated neurodegeneration leads to Alzheimer’s disease and significant dementia, which in turn contributes to deteriorating oral health and dysbiosis in gut microbiota (Paudel et al. 2022). Different oral diseases, such as the gingiva, periodontal ligament, and alveolar bone, are associated with different clinical signs of Parkinson’s, cognitive, and Alzheimer’s diseases (Kambara et al. 2025). Periodontitis is significantly correlated with systemic inflammation and has been associated with numerous systemic illnesses, including diabetes mellitus, cardiovascular disease, and rheumatoid arthritis. Despite research on the impact of psychological stress on the oral microbiome, a thorough understanding of the dental-brain axis is still deficient (Adams et al. 2017).

## Composition and diversity of the oral microbiota

### Composition of the oral microbiota

*Phyla Actinobacteria*, *Bacteroidetes*,* Chlamydia*, *Euryarchaeota*,* Fusobacteria*, *Firmicutes*, *Proteobacteria*, *Spirochaetes*, and *Tenericutes* are the most numerous in the oral cavity, which is home to almost a thousand different kinds of bacteria. It also includes several possible divisions and phyla that are not as well known, such as WPS-2, SR1, TM7, *Synergistetes*, *Chloroflexi*, and *Chlorobi*. Some of them are classified as CPR, including GN02, SR1, and TM7. A number of oral illnesses, including periodontitis and halitosis, have been associated with changes in the composition and function of the microbiome, which in turn are shaped by members of the oral CPR. Nevertheless, only TM7 had been effectively cultivated from the human oral cavity due to the difficulty in isolating pure cultures of CPR members (Dewhirst et al. 2010; Griffen et al. 2012; Camanocha and Dewhirst 2014; Bor et al. 2019, 2020).

When compared to oral bacteria, oral archaea are far less abundant and varied. While it was previously believed that these organisms could only produce methane, new research has found DNA sequences from archaea that do not produce methane in the mouth (Wade 2013; Dame-Teixeira et al. 2020). Despite their presence in caries biofilm samples, subgingival biofilms from individuals with peri-implantitis and periodontitis, and inflammatory pulp tissue, archaea are currently thought to be non-pathogenic. These findings suggest that archaea may play a role in the pathogenic mechanisms of mouth disorders; nevertheless, the pathogenicity of archaea requires more investigation (Efenberger et al. 2015; Aleksandrowicz et al. 2020; Dame-Teixeira et al. 2020).

*Aspergillus*, *Aureobasidium*,* Candida*, *Cladosporium*,* Cryptococcus*,* Fusarium*, *Gibberella*, *Penicillium*, *Rhodotorula*, *Saccharomycetales*, and *Schizophyllum* are frequent genera among the approximately 100 species of fungi found in the mouth. Probably, participant features and the low frequency of fungi in other locations contribute to the fact that fungi constitute only 0.004% of the total oral microorganisms and are mostly detected in samples taken from the hard palate, supragingival plaque, and oral rinses (Peters et al. 2017; Caselli et al. 2020). Nonetheless, new studies have shown a wide variety of fungi in saliva samples, with two separate genus-level communities identified: *Malassezia* and *Candida*. It is worth noting that samples of oral rinse from healthy persons show a great deal of individual variation in oral fungus species (Ghannoum et al. 2010; Hong et al. 2020).

Eukaryotic viruses and phages make up the oral virome. Phages are more varied and have been researched for their capacity to lyse bacteria, making them possible remedies for bacterial illnesses; eukaryotic viruses mostly comprise the Anelloviridae, Herpesviridae, and Papillomaviridae families (Shen et al. 2018; Caselli et al. 2020; Chen et al. 2020). Research has shown that many viruses identified in the mouth may stay around for a long period, rather than being transient inhabitants. The possibility that viruses in saliva might transport harmful genes to the mouth has also been suggested. We now know that oral viruses are unique to each individual, last for a long time, and are gender specific; furthermore, viral communities within a given environment tend to be quite similar and frequently even related (Pride et al. 2012; Robles-Sikisaka et al. 2013; Abeles et al. 2014).

### Diversity of the oral microbiome

Previous studies that looked at the oral microbiome mostly employed 16S rRNA gene amplicon sequencing, which is mainly used to study bacteria. More than 700 bacterial species, mostly belonging to a handful of genera in seven phyla (*Actinomycetota*, *Bacteroidota*, *Firmicutes*,* Fusobacteria*, *Pseudomonadota*, *Saccharibacteria*, TM7, and *Spirochaetota*), have been identified through these investigations. Generally speaking, people’s oral microbiomes consist of the same major bacterial species. Individuals can be distinguished from one another by their genetic diversity, which is mostly attributable to variances in the relative abundances of various taxa, strain-level changes, and the occurrence of unusual strains (Escapa et al. 2018; Tierney et al. 2019; Oren and Garrity 2021).

Oral microbiome research has made more use of metagenomic and metatranscriptomic sequencing thanks to falling sequencing prices, improved computer capacity, and new bioinformatics tools. Not only does metagenomics enable the discovery of species lacking 16S rRNA genes, but it also significantly broadens the known oral microbiome to encompass a vast array of viruses, fungi, protozoa, and archaea, among other less frequent but relevant taxa (Li et al. 2022a; Baker et al. 2024).

## Role of oral microbiota metabolites in health

Recent advancements in metabolomics and microbiome research have highlighted the critical role of microbial metabolites, which are defined as small molecules generated during microbial metabolic activity, in maintaining oral and systemic health (Cambeiro-Pérez et al. 2018). These metabolites are not merely metabolic waste; rather, they function as bioactive molecules, participating in host–microbe and microbe–microbe interactions that influence immune modulation, inflammation, tissue integrity, and disease susceptibility (Luo et al. 2024).

Among the most studied are short-chain fatty acids (SCFAs), particularly acetate, propionate, and butyrate, which are produced by anaerobic fermentation of dietary carbohydrates by oral bacteria such as *Prevotella*,* Fusobacterium*, and *Veillonella*. In physiological conditions, SCFAs contribute to the maintenance of mucosal integrity, exert anti-inflammatory effects, and modulate the function of local immune cells (Nataraj et al. 2020). Butyrate (Oh et al. 2022), for example, promotes epithelial differentiation and regulates inflammatory pathways by acting on histone deacetylase activity. However, in diseased states like periodontitis, elevated SCFA concentrations can become cytotoxic, leading to tissue breakdown and immune evasion (Xavier-Santos et al. 2022).

Antimicrobial peptides (AMPs) are peptides that create gaps in bacterial membranes or impede the synthesis of the bacterial wall. The AMP synthesis may occur autonomously or in response to an infection. They serve as endotoxin neutralizers and immunomodulatory agents to enhance innate immunity (Aggarwal et al. 2022). Antimicrobial peptides synthesized by genetically modified *Lactococcus lactis* inhibit the proliferation of enterococci and multidrug-resistant *Enterococcus faecium* strains in the digestive tract (Liang and Xing 2023). These features enable AMPs to engage with many bacterial membrane components, including teichoic acids. The mechanism of action involves electrostatic interactions between positively charged AMP amino acids and the negatively charged lipopolysaccharides of bacterial membranes (Ayad et al. 2025).

Another critical group of metabolites includes nitrogen and sulfur-containing compounds such as ammonia, hydrogen sulfide (H₂S), and methyl mercaptan, often resulting from proteolytic metabolism by species like *Porphyromonas gingivalis* and *Treponema denticola*. While some of these molecules act as signaling agents involved in redox balance and vasodilation, their accumulation is associated with inflammatory responses, cellular toxicity, and tissue damage (Martyniak et al. 2025). H₂S, for instance, interferes with mitochondrial function, disrupts cell signaling, and induces oxidative stress, thereby contributing to the chronic inflammation observed in periodontal diseases.

Beyond their local effects, microbial metabolites produced in the oral cavity can enter systemic circulation and exert distal effects (Rosier et al. 2021). One of the most prominent examples of this oral-systemic link is the nitrate–nitrite–nitric oxide pathway. Specific oral bacteria, such as *Neisseria* and *Rothia*, possess the enzymatic machinery to reduce dietary nitrate to nitrite, which is further converted into nitric oxide, a vasodilator with cardioprotective effects. Disruption of this microbial function, for example, through antimicrobial mouthwash use, may impair blood pressure regulation and endothelial function, highlighting the systemic implications of oral microbial activity (Li et al. 2022b).

Recent studies have also emphasized the immunomodulatory effects of microbial metabolites. Tryptophan-derived indole compounds produced by oral bacteria activate the aryl hydrocarbon receptor (AhR) pathway in host cells, influencing the differentiation of T-helper cells and the production of antimicrobial peptides. Such interactions are essential for maintaining mucosal immunity and preventing overactivation of inflammatory responses. Disruption of these pathways has been implicated in systemic inflammatory conditions, including cardiovascular disease and metabolic syndrome (Ghasemi 2024; Lu et al. 2024; Mafe and Büsselberg 2025).

Moreover, polyamines such as spermidine and putrescine, produced through amino acid decarboxylation, play a dual role in health and disease (Wang et al. 2024). These molecules are essential for cell growth, DNA stabilization, and epithelial repair, yet excessive production has been linked to tumorigenesis and persistent inflammation. Elevated polyamine levels in the oral cavity have been observed in patients with oral squamous cell carcinoma and advanced periodontal disease, suggesting that microbial metabolic outputs may contribute to oncogenic processes (Sakanaka 2024).

Advances in salivary metabolomics have further elucidated the metabolic signatures associated with chronic oral diseases (Nataraj et al. 2020). In periodontitis, distinct shifts in salivary metabolites include increased levels of amino acids, lipids, and polyamines which have been identified. These metabolic alterations reflect underlying microbial shifts and may also provide insight into disease mechanisms and progression. Importantly, these profiles hold promise for developing non-invasive diagnostic tools for early detection and monitoring of oral and systemic diseases (Albahri et al. 2025).

The impact of oral microbial metabolites is not limited to host–microbe interactions but also extends to microbe–microbe communication. Through interspecies signaling, metabolites regulate biofilm structure, microbial competition, and resistance to external stressors. For example, metabolic byproducts such as lactic acid or hydrogen peroxide can inhibit the growth of competing species, shaping community composition and stability. Disruption of these interactions, often due to external factors like diet, antibiotics, or oral hygiene practices, may destabilize the oral ecosystem and predispose to disease (Giordano-Kelhoffer et al. 2022).

From a clinical perspective, the modulation of oral microbial metabolism presents new avenues for therapy (Basic and Dahlén 2023). Approaches such as the use of prebiotics to enhance beneficial metabolite production or the application of selective antimicrobials that target pathogenic metabolic pathways are gaining interest. Additionally, probiotic strains capable of restoring a healthy metabolic balance are being explored in both the prevention and treatment of oral and systemic conditions. These interventions, guided by individual metabolic and microbial profiles, hold the potential to shift the paradigm toward personalized oral healthcare (Wang et al. 2024).

## Imbalance of oral microbiota in oral disorders

### Dental caries

Dental caries represents one of the most prevalent chronic diseases globally, fundamentally rooted in the disruption of oral microbial homeostasis rather than simple bacterial infection. This complex multifactorial disease emerges from microbiome dysbiosis characterized by the proliferation of acid-producing and acid-tolerant bacterial species, particularly mutans streptococci, lactobacilli, and emerging pathogens like *Scardovia wiggsiae* (Zhan 2018). The pathogenesis involves a dynamic interplay between cariogenic bacteria, dietary carbohydrates, and host factors, ultimately leading to demineralization of tooth structures through localized acid production within dental biofilms (Struzycka 2014). Understanding this ecological imbalance has revolutionized our approach to caries prevention and treatment, shifting focus from purely restorative interventions to microbiome-targeted therapeutic strategies that aim to restore microbial equilibrium in the oral cavity (Luo et al. 2024).

#### Fundamental mechanisms of oral microbiome dysbiosis

The transition from health to disease involves multiple stages of microbial community restructuring, each characterized by specific bacterial populations and metabolic activities. Initially, the oral microbiota exists in a dynamic stable stage where plaque formation occurs naturally on tooth surfaces, providing protection against exogenous pathogens while maintaining mineral balance through controlled acid production and neutralization (Marsh 1994). This protective biofilm contains mainly beneficial bacteria that produce mild and infrequent acidification, allowing the natural remineralization processes to predominate over demineralization. However, when environmental pressures such as frequent sugar exposure occur, this stability breaks down, initiating a cascade of microbial changes that ultimately lead to caries development (Struzycka 2014).

#### Primary cariogenic bacteria and their pathogenic mechanisms

*Streptococcus mutans* stands as the most extensively studied and widely recognized cariogenic bacterium, representing the archetypal pathogen in dental caries development. This organism possesses several key virulence factors that enable it to thrive in the acidic environment it creates, including exceptional acid tolerance, efficient acid production from dietary sugars, and strong biofilm-forming capabilities (Baker et al. 2024). Elevated levels of *S. mutans* have been consistently detected in subjects before caries development, and high concentrations are observed in dental plaque and saliva of individuals with active decay (Zhan 2018). The organism’s ability to metabolize sucrose and other fermentable carbohydrates into lactic acid creates localized pH drops that demineralize tooth enamel, while its acid tolerance allows it to survive and continue thriving in the harsh acidic environment it generates (Kressirer et al. 2017).

The pathogenic potential of these bacteria extends beyond their characteristics to include their complex interactions within multispecies biofilms. Recent fluorescence microscopy studies have revealed that *S. mutans* can adopt different spatial organizations within biofilms, including a “rotund” architecture where the bacteria are densely packed within an extracellular scaffold (Baker et al. 2024). When organized in this configuration, *S. mutans* generates more severe localized acidification and causes dramatically increased demineralization of underlying enamel surfaces compared to when the bacteria are dispersed throughout the biofilm. This spatial organization demonstrates how the three-dimensional structure of microbial communities directly influences their pathogenic potential and highlights the importance of understanding biofilm architecture in caries development (Baker et al. 2024).

#### Ecological stages and mechanisms of dysbiosis development

The development of caries-associated microbiome dysbiosis follows a predictable ecological progression that can be understood through the lens of environmental adaptation and microbial selection pressures. Takahashi and Nyvad proposed a comprehensive three-stage model that describes the transition from oral health to disease, beginning with a dynamic stable stage on sound enamel surfaces. In this initial stage, dental plaque contains primarily beneficial bacteria such as non-mutans streptococci and *Actinomyces* species that maintain mild and infrequent acidification patterns, allowing the natural balance between demineralization and remineralization to favor net mineral gain. This protective equilibrium can persist indefinitely under appropriate conditions, with the oral microbiota serving as a barrier against pathogenic colonization (Zhan 2018).

The second stage, termed the acidogenic stage, represents the critical transition point where the increasing frequency of sugar and fermentable carbohydrate consumption begins to alter the microbial ecosystem. During this phase, the resident non-mutant bacteria undergo aciduric and acidogenic adaptation, developing enhanced capabilities for acid production and tolerance (Zhan 2018). Simultaneously, selective pressure favors the proliferation of naturally aciduric bacterial species, creating a gradual shift in community composition toward more cariogenic organisms. This stage is characterized by increasingly frequent pH drops in dental plaque, shifting the demineralization–remineralization balance toward net mineral loss and initiating the earliest stages of enamel breakdown (Struzycka 2014; Zhan 2018).

The final aciduric stage represents the establishment of a fully dysbiotic microbial community dominated by highly acidogenic and acid-tolerant bacteria. Prolonged exposure to acidic conditions creates an environment that strongly selects specialized organisms such as mutans streptococci, lactobacilli, and aciduric non-mutans streptococci. At this stage, the microbial community has undergone fundamental restructuring, with pathogenic species becoming predominant and the protective functions of the original commensal bacteria being largely lost. The biofilm environment becomes chronically acidic, creating conditions that favor continued bacterial acid production and sustained demineralization of tooth structures (Struzycka 2014; Zhan 2018).

#### Biofilm formation and spatial organization in caries development

The formation and maturation of dental biofilms represent critical processes in the development of caries-associated microbiome dysbiosis, as these three-dimensional microbial communities create unique microenvironments that facilitate pathogenic bacterial activities. Dental plaque functions as a classic biofilm, exhibiting features of organized bacterial communities embedded within self-produced extracellular matrices that provide protection from environmental stresses and antimicrobial agents (Marsh 1994). Biofilm architecture creates distinct microenvironments with varying oxygen tensions, pH levels, and nutrient availability, allowing different bacterial species to occupy specialized niches within the same community. This spatial organization enables the coexistence of diverse bacterial populations and facilitates metabolic cooperation between species with complementary functions (Rosier et al. 2014; Zhu et al. 2022).

Recent advances in fluorescence microscopy and molecular imaging have revealed the sophisticated spatial organization within cariogenic biofilms and their direct relationship to pathogenic potential. Studies utilizing fluorescence in situ hybridization (FISH) techniques have demonstrated that the distribution of bacterial species within biofilms is not random, but rather follows specific patterns that optimize acid production and tolerance (Baker et al. 2024). For example, *S. mutans* can adopt different architectural arrangements within biofilms, including dispersed patterns throughout the biofilm matrix or concentrated “rotund” formations where bacteria cluster densely within extracellular scaffolds. When *S. mutans* organizes in the rotund architecture, it generates significantly more severe localized acidification and causes dramatically increased demineralization of underlying enamel surfaces compared to dispersed arrangements (Baker et al. 2024).

The biofilm matrix itself plays a crucial role in maintaining the acidic microenvironment necessary for sustained demineralization. Extracellular polysaccharides produced by cariogenic bacteria, particularly glucans synthesized by *S. mutans* from sucrose, create a protective scaffold that retains bacterial acids in close contact with tooth surfaces (Yama et al. 2023). This matrix also limits the diffusion of buffering agents from saliva, creating isolated microenvironments where pH can remain low for extended periods despite the presence of natural neutralizing mechanisms in the oral cavity. Additionally, the biofilm matrix facilitates bacterial adhesion and provides structural stability that allows the microbial community to resist mechanical removal by normal oral functions such as salivation and tongue movement (Marsh et al. 2015).

The development of dental biofilms involves complex temporal dynamics that influence both microbial composition and pathogenic potential. Initial colonization occurs through the binding of pioneer bacterial species to the acquired pellicle, a proteinaceous layer that forms on clean tooth surfaces within minutes of exposure to the oral environment (Spatafora et al. 2024). Early colonizers, typically streptococci and *Actinomyces* species, establish the foundation for subsequent microbial succession, with later colonizing species building upon the established bacterial community. Recent research has revealed that bacterial aggregates in saliva can bind to teeth as large, multi-species units rather than individual cells, providing a growth advantage and helping explain why late colonizers can be detected as soon as 30 min after mechanical biofilm removal. This aggregate binding mechanism suggests alternative pathways for biofilm development and highlights the resilience of oral microbial communities in reestablishing themselves following disruption (Spatafora et al. 2024).

### Periodontal disease

**Fig. 1:**
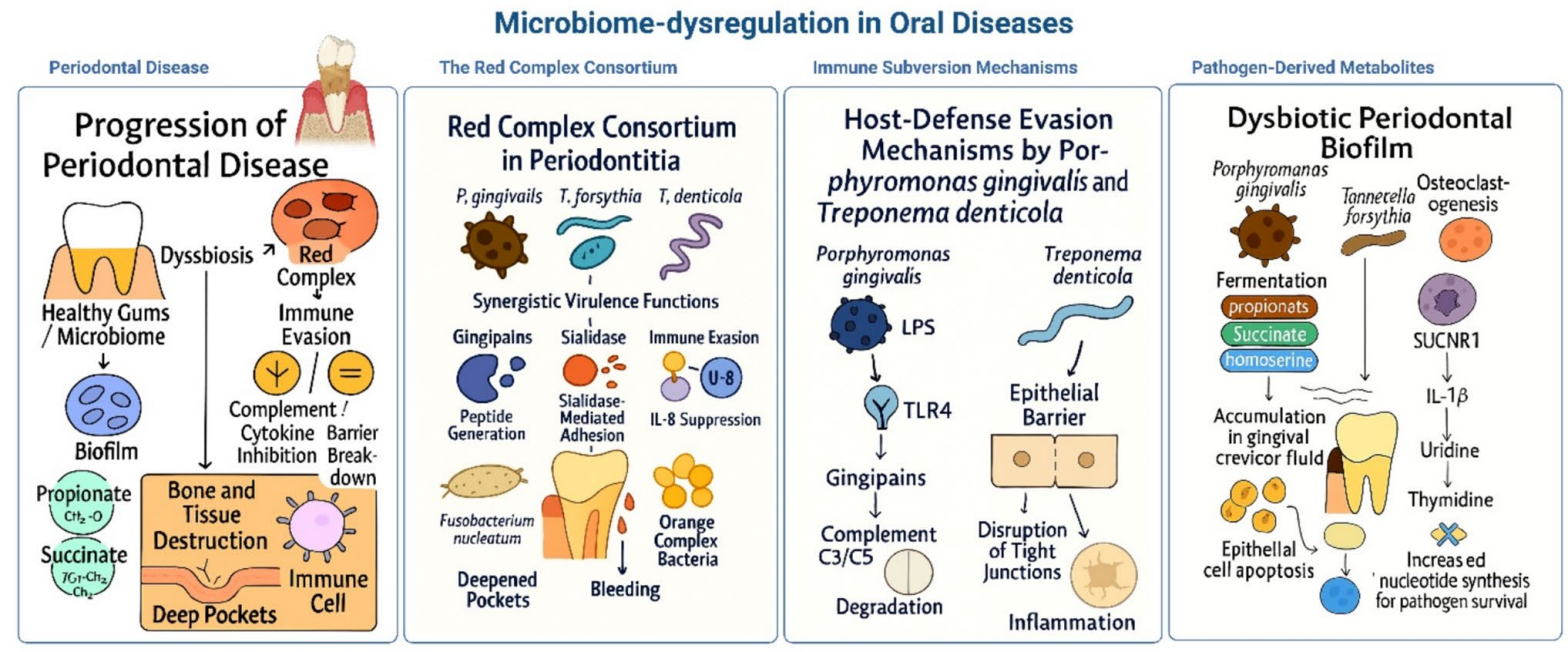
Disruption of the oral microbiome drives periodontal disease through red complex synergy, immune evasion, and metabolite-induced inflammation, leading to tissue destruction and deepened periodontal pockets. LPS, lipopolysaccharide; TLR4, Toll-like receptor 4; C3, Complement 3; SUCNR1, succinate receptor 1; IL-1β, interleukin 1β

Periodontal diseases, including gingivitis and periodontitis, represent a significant global health burden characterized by inflammation-driven destruction of the periodontal ligament and alveolar bone. Emerging research underscores the central role of oral microbiota dysbiosis in initiating and perpetuating these conditions (Giordano-Kelhoffer et al. 2022). Here, we shed light on the transition from a health-associated oral microbiome to a dysbiotic state, the virulence mechanisms of keystone pathogens, the metabolic interplay driving tissue destruction, and the challenges in achieving microbial equilibrium post-treatment (Fig. 1).

#### The red complex consortium

The red complex—*P. gingivalis*, *T. forsythia*, and *Treponema denticola* (*T. denticola*)—emerge as a critical polymicrobial consortium in periodontitis progression. These anaerobes exhibit synergistic relationships: *P. gingivalis* produces gingipains that cleave host proteins into peptides utilized by *T. denticola*, while *T. forsythia*’s sialidase activity exposes epithelial cell receptors for adhesion (Mohanty et al. 2019). Clinically, red complex abundance correlates with pocket depth, bleeding on probing, and radiographic bone loss. Notably, *P. gingivalis* operates as a keystone pathogen, subverting host immunity at low abundance to enable dysbiosis. By inhibiting neutrophil chemotaxis via C5a receptor cleavage and suppressing IL-8 production, it creates a permissive environment for secondary colonizers like *Fusobacterium nucleatum* and orange complex bacteria (Abdulkareem et al. 2023) (Fig. 1).

#### Immune subversion mechanisms

*Porphyromonas gingivalis* employs sophisticated strategies to manipulate host defenses. Its lipopolysaccharide (LPS) exhibits weak TLR4 agonism, blunting proinflammatory cytokine responses, while gingipains degrade complement components (C3, C5) and antimicrobial peptides (LL-37). This dual action paralyzes bacterial clearance while sustaining low-grade inflammation that fuels tissue breakdown. Moreover, *T. denticola*’s chymotrypsin-like protease (dentilisin) disrupts epithelial tight junctions, facilitating bacterial invasion and systemic dissemination (Mohanty et al. 2019; Abdulkareem et al. 2023) (Fig. 1).

#### Pathogen-derived metabolites

Dysbiotic biofilms alter the metabolic landscape of the periodontal pocket. *P. gingivalis* and *T. forsythia* ferment amino acids into propionate, succinate, and homoserine, which accumulate in the gingival crevicular fluid. These metabolites induce epithelial cell apoptosis, stimulate osteoclastogenesis via RANKL upregulation, and impair neutrophil phagocytosis. Notably, succinate activates the SUCNR1 receptor on macrophages, triggering IL-1β release and amplifying inflammatory bone resorption (Ishihara et al. 2025). Concurrently, pyrimidine metabolism shifts favor pathobiont survival: elevated uridine and thymidine in diseased sites enhance microbial nucleotide synthesis, while depleted thymine correlates with *P. gingivalis* prevalence (Yama et al. 2023; Ishihara et al. 2025) (Fig. 1).

### Halitosis

Halitosis is an unpleasant odor in the mouth. There are several underlying causes of halitosis, including, but not limited to, dental problems, dryness of the mouth, oral infections, some medications, or other extra-oral health issues (Li et al. 2023). This unpleasant bad smell from the mouth comes from the exhalation of volatile sulfur compounds (VSCs) that are produced by Gram-negative microorganisms in the oral cavity through their proteolytic degrading enzymes. These VSCs are hydrogen sulfide, methyl mercaptan, and dimethyl sulfide (Izidoro et al. 2022).

Halitosis can be classified based on the International Society for Breath Odor Research (ISBOR) into genuine halitosis and delusional halitosis. Genuine halitosis can be further classified into physiological and pathological (oral and extra-oral) halitosis. On the other hand, the delusional or false halitosis can be pseudo-halitosis and halitophobia (Viana et al. 2024).

#### Genuine or true halitosis

This type can be defined as long-term persistent halitosis, which can be noticed by others. Physiological halitosis is mainly caused by the decomposition of organic substances in the mouth without any detected oral medical conditions, such as the smell of the mouth when getting up from sleep. It is usually transient and can result from cigarette smoking, anxiety, or other psychological disorders (Mento et al. 2021).

The extra-oral pathological halitosis can be any medical issue originating outside of the oral cavity, including, but not limited to, respiratory tract infections, gastrointestinal and metabolic disorders, and systemic diseases (Izidoro et al. 2022). Some medications also contribute to the development of halitosis by causing dry mouth, such as anticholinergics, antihistamines, and antipsychotics (Oki and Salsabila 2024). Other medications categorized as medication-related osteonecrosis of the jaw (MRONJ), for instance, bisphosphonates, were also reported to cause halitosis (Choi et al. 2024).

**Fig. 2:**
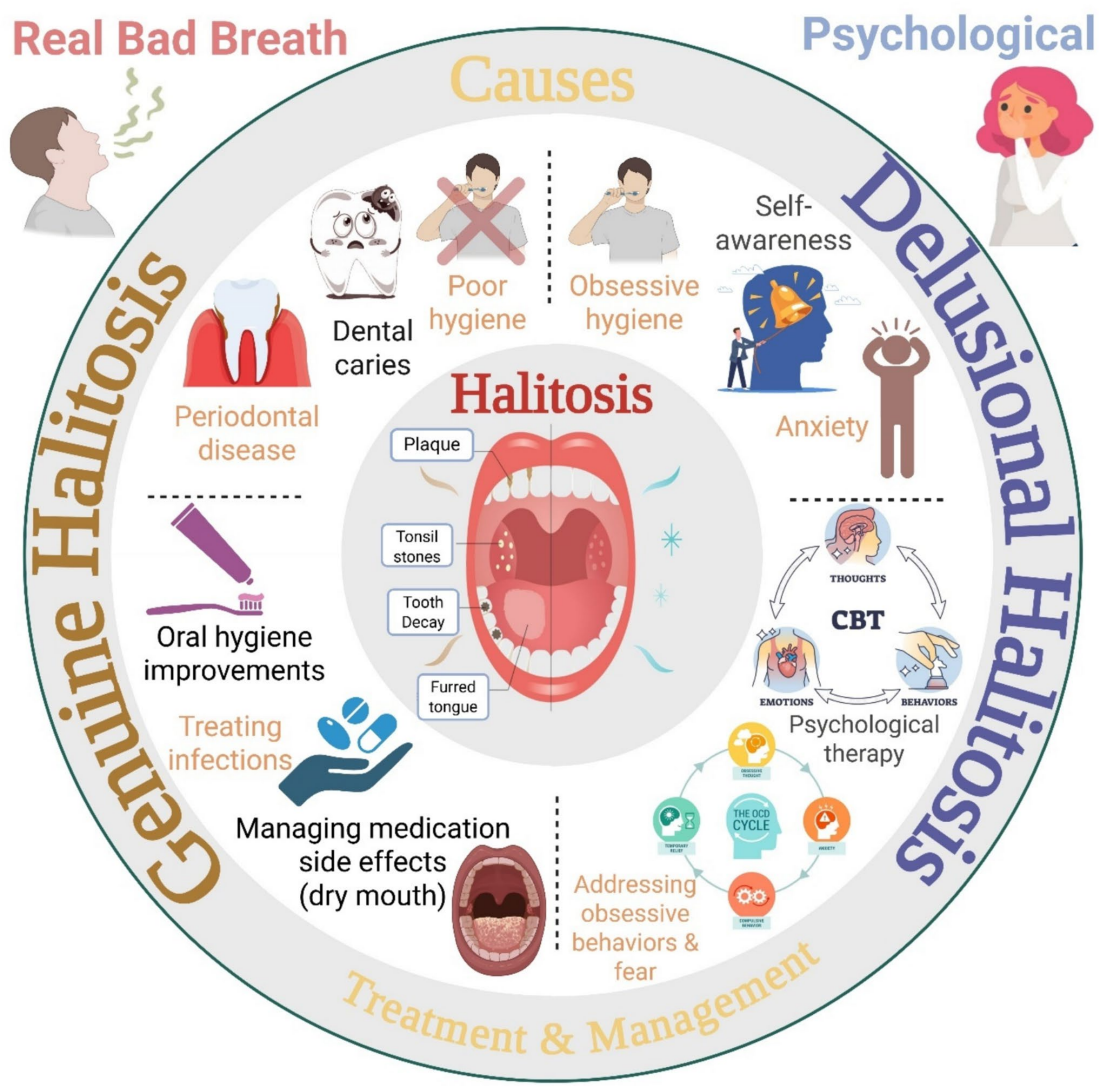
Illustration of halitosis types, causes, and management strategies, distinguishing between genuine and delusional halitosis. CBT, cognitive-behavioral therapy

Regarding the oral causes of halitosis, they represent the major source of halitosis. Odontogenic halitosis results from bad oral hygiene, dental caries, or the accumulation of food debris in the mouth. Tongue coating, especially the dorsoposterior region, is problematic because of its papillary structure, which encourages the accumulation of microorganisms, and also due to the difficult accessibility of hygienic tools to this area of the tongue (Bicak 2018). It is worth remarking that tongue coating largely contributed to the production of methyl mercaptan as the main VSC (Mogilnicka et al. 2020). Periodontal disease, which involves gingivitis and periodontitis, also contributes to the malodor of the mouth. This form of halitosis is attributed to the damaged oral epithelium, increased bacterial load, increased acidity of the mouth, and decreased oxygen tension concomitantly with the formed pockets. All these factors exacerbate the production of VSCs. Nevertheless, some studies deny periodontal disease as being the culprit in the development of halitosis, considering it as closed pockets (Renvert et al. 2020). Oral candidiasis and oral cancer were also implicated in the development of halitosis (Izidoro et al. 2022) (Fig. 2).

#### Delusional or false halitosis

Delusional halitosis is a condition in which individuals though they have halitosis, but they do not, and this is mainly due to obsessive care about hygiene and self-awareness, or psychological illness (Tsuruta et al. 2017). In pseudo-halitosis, the person is convinced that they have halitosis even though it is not noticed by others or detected by a dentist (Kim et al. 2021), whereas halitophobia is the extreme fear of having halitosis, especially after recovery from previously diagnosed halitosis episodes, and this type can fall under olfactory reference syndrome (ORS) (Takenoshita et al. 2021) (Fig. 2).

#### Oral microbiota and halitosis

It has been reported that the oral cavity is the second microenvironment with a large population of microbiomes after the colon (Mok et al. 2017). The principal oral microbiota consists of phyla including *Firmicutes*, *Proteobacteria*,* Fusobacteria*, *Bacteroidetes*, and *Actinobacteria* (Hampelska et al. 2020). Several bacterial species have been linked to the development of halitosis, mainly *Peptostreptococcus* spp., *Alloprevotella* spp., *Eubacterium* spp.,* Campylobacter* spp., *Stomatobaculum*, and *Granulicatella*, which are VSC producers. Interestingly, the bacterial species that cause halitosis in healthy individuals differ from those that cause halitosis in patients with periodontitis. Therefore, detecting the type of microbiota can help distinguish healthy from pathological states of the oral cavity (Li et al. 2023). For example, *P. gingivalis*,* Treponema denticola*,* T. forsythia*, and different *Capnocytophaga* species were found in periodontitis-associated halitosis but not in the halitosis developed in healthy individuals. Accordingly, this can be useful in excluding periodontitis as the cause for halitosis (Foo et al. 2021).

Hydrogen sulfide is mainly released by the effect of bacterial species, such as *Bacteroides intermedius*, *Bacteroides* spp., and *Capnocytophaga ochracea* on L-cysteine, whereas other species like *Bacteroides gracilis*,* B. intermedius*,* B. loescheii*,* B. oralis*, *Eubacterium lentum*, and *Eubacterium* spp. produce it from the serum. *Eubacterium* spp.,* F. nucleatum*, and *F. periodonticum* produce methyl mercaptan from L-methionine, whereas *P. endodontalis*,* P. gingivalis*, and *T. denticola* release it from the serum (Hampelska et al. 2020). It is worth mentioning that *Streptococcus*, *Prevotella*, *Rothia*, *Actinomyces*, *Granulicatella*,* Neisseria*, *Terrahaemophilus*, *Veillonella*, *Gemella*,* Fusobacterium*, and *Haemophilus* were almost equally detected in the salivary secretion from both intra-oral halitosis and healthy controls (Foo et al. 2021). Intriguingly, most of the implicated bacterial species in halitosis have similar enzymes that are encoded by common genes (*megL*, *lcs*, *mgl*). These enzymes are methionine gamma lyase, L-cysteine sulfide-lyase, and L-methioninase. It is interesting to note that some food like onion, garlic, cabbage, and mushrooms provide more substrates for bacterial reductases, thereby increasing the production of hydrogen sulfide and worsening the malodor of the mouth (Mogilnicka et al. 2020).

However, halitosis induced by microbiota imbalance is a complicated phenomenon and cannot be attributed to a single species because these microorganisms create multiple and intercalated metabolic pathways for their survival. The co-presence of multiple bacterial communities was identified by using terminal-restriction fragment length polymorphism (TRFLP), which revealed saccharolytic Gram-positive bacteria with the formerly identified saccharolytic or proteolytic Gram-negative bacteria in the oral environment (Foo et al. 2021). Another Gram-positive *Solobacterium moorei* was detected in high amounts in a patient with halitosis (Wen et al. 2016). *Porphyromonas gingivalis*,* P. endodontalis*, and* F. nucleatum* are VSCs producers, but these species were detected in high loads in patients with halitosis compared to normal healthy individuals. These findings suggest that the released VSCs are exacerbated by interactions with other coexisting bacterial communities (Takeshita et al. 2012). Notwithstanding, integrative metabolomic and meta-transcriptomic studies would be more beneficial in pinpointing the underlying cause of halitosis. Additionally, in vitro co-cultivation experiments could clarify whether the bacterial taxa directly contribute to the development of halitosis or influence other species through the formation of interactive networks. These approaches have to be considered together with the quantitative measures of the implicated bacterial populations rather than just the qualitative determination of the phyla.

### Oral candidiasis

*Candida* spp. is the predominant fungus that is normally found within the oral microbiome community. Oral candidiasis, or oral thrush, is an opportunistic fungal infection of the mouth. It is primarily caused by *Candida albicans*, although other species can also contribute to the disease to a lesser extent (Tiwari and Dangore-Khasbage 2024). Oral thrush largely affects the tongue, but other mucosal areas within the mouth can also be affected. This thrush appears as white patches, redness, soreness, and, in complicated cases, can even cause trouble in swallowing (Rajasekaran et al. 2024).

#### Pathogenesis

**Fig. 3:**
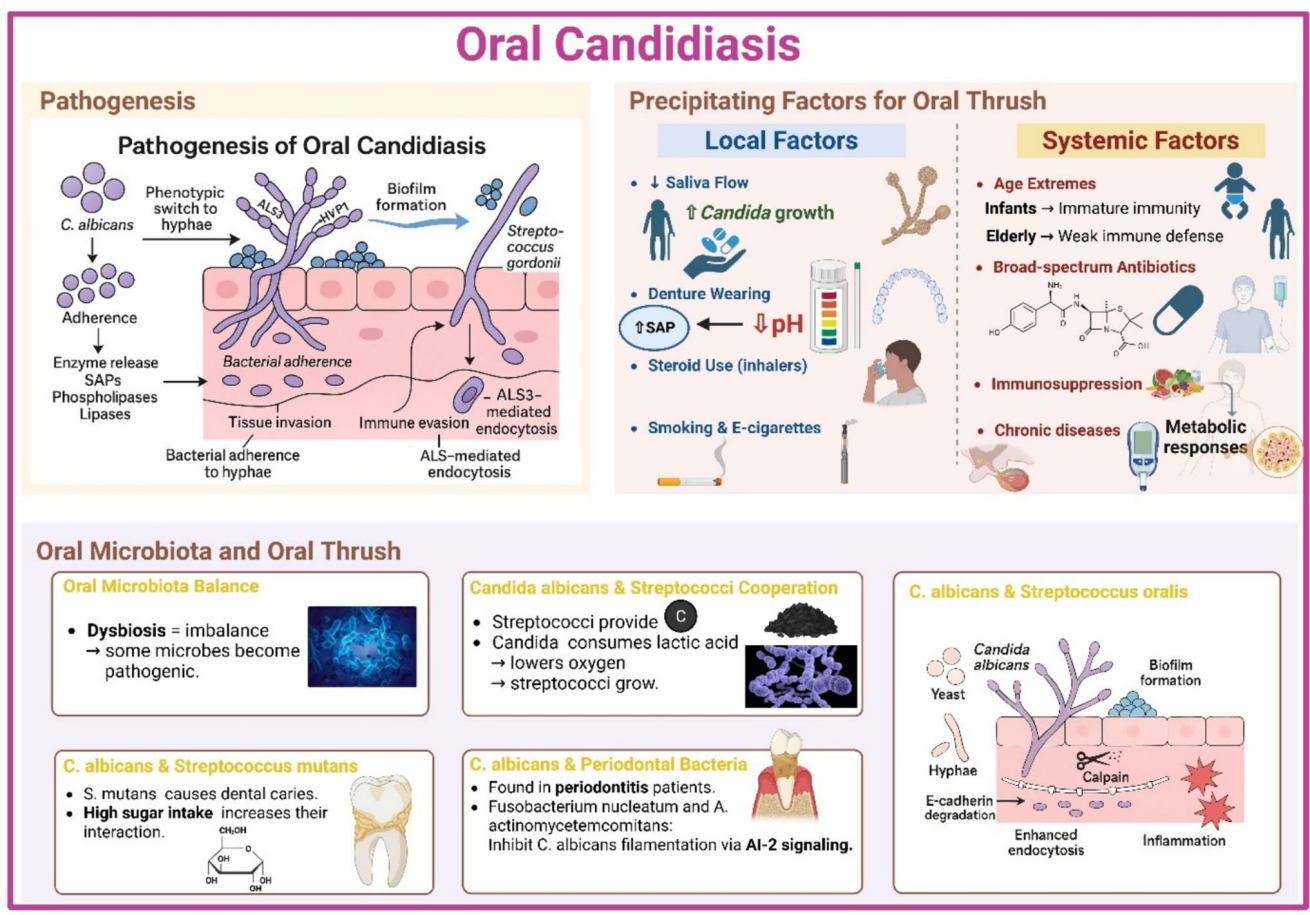
Overview of the pathogenesis, precipitating factors, and microbial interactions involved in oral candidiasis. ALS, amyotrophic lateral sclerosis

The pathogenicity of oral candidiasis is intricate and involves multiple aspects. *C. albicans* needs first to adhere to the epithelial surface through reversible hydrophobic and electrostatic interactions, which can be disabled by salivary flow and swallowing. This attachment is mediated by specific receptors on the host’s oral cavity, such as agglutinin-like sequence (ALS), mainly ALS3, and hyphal wall protein (HWP), mainly HWP1 glycoproteins. ALS3 also plays a role in bacterial adherence to the hyphae of *C. albicans* (Vila et al. 2020). Then, the fungus undergoes a phenotypic switch from the yeast form to the hyphal form and initiates fungal–bacterial interactions, which form the biofilms. Maliciousness of the oral thrush and resistance to the therapies are largely linked to the formed biofilms owing to the involved bacterial populations (Rajasekaran et al. 2024). For example, *C. albicans* interacts with *Streptococcus gordoni*, a member of oral microbiota, via cross-linking between ALS3 and the cell wall–associated protein SspB on the bacteria, indicating the complexity of microbiota colonization and its implication in the oral diseases involving the thrush (Silverman et al. 2010) (Fig. 3).

In order to penetrate the tissue, *C. albicans* must retain its hyphal form. This invasion destroys the epithelial cells, induces inflammation, and forms the observed white patches. These events are mediated through the release of hydrolytic enzymes, like secreted aspartyl proteinases (SAPs), phospholipases, and lipases. Unfortunately, these enzymes impair the host defense mechanisms involving the released antibodies and antimicrobial peptides (Rajasekaran et al. 2024). Moreover, candidalysin is a toxin released by *C. albicans* that triggers an immune response and ultimately induces tissue destruction (Swidergall et al. 2019). One more pathogenic tactic is endocytosis to epithelial cells, which is mediated through ALS3 and degradation of E-cadherin (Phan et al. 2007).

#### Precipitating factors for oral thrush

The host immune state and other microbiota members are the main factors that impact the growth of *Candida*. Therefore, reduced immunity, immune diseases, and some other medical issues, or certain medications, can disturb the balanced normal flora in the mouth and stimulate *Candida*’s overgrowth, causing it to act like a pathogen (Lu 2021). Generally, these factors can be classified into local and systemic factors.

#### Local factors

Salivary dysfunction and reduced amount of the released antimicrobial agents are highly contributing to oral candidiasis. This is commonly observed in elderly people, polypharmacy, and immunocompromised patients (Vila et al. 2020). Prolonged denture wearing is also a common local factor, which triggers the thrush through making the oral cavity more acidic, rendering the environment more suitable for the activity of SAP (Schaller et al. 2005). Chronic use of corticosteroid therapy, even the inhalers, can cause oral thrush by diminishing immunity. Additionally, tobacco smokers were identified as carriers of oral candidiasis (Vila et al. 2020). Intriguingly, even electronic nicotine delivery systems were found to contribute to the development of thrush. A recent study suggested that E-cigarette induces *C. albicans* proliferation and modulate its interaction with epithelial cells through upregulation of SAP2, SAP3, and SAP9 genes (Alanazi et al. 2019) (Fig. 3).

#### Systemic factors

Elderly people and also infants are at high risk for developing oral candidiasis owing to the diminished immunity or immature defense mechanisms, respectively (Vila et al. 2020). Broad-spectrum antibiotics induce an imbalance in the oral microbiota, a condition that encourages the growth of *C. albicans* (Baumgardner 2019). Immunocompromising conditions, immunosuppressive agents, chemotherapies, radiation therapy, nutritional disorders, and certain endocrine diseases are also major risk factors for developing oral thrush (Vila et al. 2020) (Fig. 3).

#### Oral microbiota and oral thrush

Under normal conditions, the oral microbiota communities are balanced to maintain oral health (Duran-Pinedo and Frias-Lopez 2015). When this ecosystem is disturbed, certain members become pathogens, and this is called dysbiosis. This unfavorable condition leads to the development of multiple oral diseases. It is worth mentioning that oral microbiota communities act as a cohort unit rather than a solitary community with extensive synergism and intercalated metabolic pathways. For instance, the metabolites produced by a certain species may act as a nutrient for another species (O'Donnell et al. 2015).

*Candida albicans* commonly interacts with oral *Streptococci* spp. to maintain its persistence since the bacteria offer the binding sites required for the stability of *C. albicans* adhesion. Moreover, *Streptococci* provide the metabolic needs for *C. albicans*, principally the carbon source necessary for its proliferation. Harmoniously, *C. albicans* consumes the lactic acid produced by* streptococci*, thereby decreasing the oxygen tension, which favors the growth of* streptococci* (Vila et al. 2020).

*Streptococcus mutans* is a specific species with which *C. albicans* commonly interacts. *Streptococcus mutans* is believed to be the main cariogenic bacterium for developing dental caries. Therefore, *C. albicans* can be indirectly incorporated in the occurrence of dental caries (Lu et al. 2023). Supporting evidence showed that *S. mutans* frequently co-exists in dental biofilms that also contain *C. albicans*, and other evidence showed that *C. albicans* was detected in individuals with dental caries (Xiao et al. 2017; Pereira et al. 2018). Unexpectedly, increased sugar consumption favors the metabolic interactions between *C. albicans* and *S. mutans* (Brito et al. 2021). *Streptococcus oralis* is another species that also interacts with *C. albicans*. This interaction was suggested to reinforce the filamentation and the biofilm formed by *C. albicans*, leading to exaggerated inflammation and worsening of the tongue lesion (Xu et al. 2014). This dysbiosis of *C. albicans* while interacting with *S. oralis* was suggested to be mediated through hyperactivity of calpain that degrades E-cadherin in epithelial cells, and therefore, enhancing endocytosis of *C. albicans* to epithelial cells (Vila et al. 2020) (Fig. 3).

Far from dental caries, although the intermingling between *C. albicans* and periodontitis-induced bacteria, commonly *Porphyromonas gingivalis* and *Fusobacterium nucleatum*, is not fully understood, *C. albicans* was detected in patients with periodontitis, and its load was also correlated with the severity of the disease (Jabri et al. 2022). Indeed, the relationship between *P. gingivalis* and *C. albicans* is controversial; a study suggests that *P. gingivalis* promotes hyphal form and invasion of *C. albicans* (Thein et al. 2006), whereas another work claimed that *P. gingivalis* inhibits the survival of the fungus (Urzúa et al. 2008). On the other hand, *F. nucleatum* was found to have an inhibitory effect on the fungal proliferation and filamentation (Satala et al. 2022). *Aggregatibacter actinomycetemcomitans* is another species that is highly linked to severe periodontitis. This bacterium inhibits the fungal filamentation and the biofilm formation through the secretion of autoinducer 2 (AI-2) (Baker et al. 2017) (Fig. 3).

Other bacterial species are also intercalated with *C. albicans*, for example, *Staphylococcus aureus*; both coexisted in a mouse model for oral candidiasis, which developed a severe systemic bacterial infection (Schlecht et al. 2015). Furthermore, *S. gordonii* was found to enhance the formation of filamentation through the release of AI-2 (Satala et al. 2022). Nevertheless, much remains to be investigated regarding fungal–bacterial interactions and their metabolic networks, with consideration given to the influence of chemical and biological metabolites that may favor interspecies communities.

The polymicrobial interactions, including fungi and bacteria, are extremely well reported in studies trying to find the interactions between fungi and bacteria. Research utilizing saliva-coated discs demonstrated that *C. albicans* induces the expression of the *S. mutans* virulence gene glucosyltransferase B, which catalyzes the synthesis of α-glucans and adheres to mannans on the surface of *C. albicans*, hence facilitating the creation of the extracellular matrix (Hwang et al. 2017). A number of additional oral microorganisms were also examined for their interaction with *C. albicans*. In periodontal infections, *Aggregatibacter actinomycetemcomitans* and* Fusobacterium nucleatum* impede the germination of *C. albicans* in vitro through the secretion of the quorum-sensing molecule AI-2 or through physical contact, respectively (Bachtiar et al. 2014).

## Microbiome-based therapies in oral diseases

### Probiotics and postbiotics

**Fig. 4:**
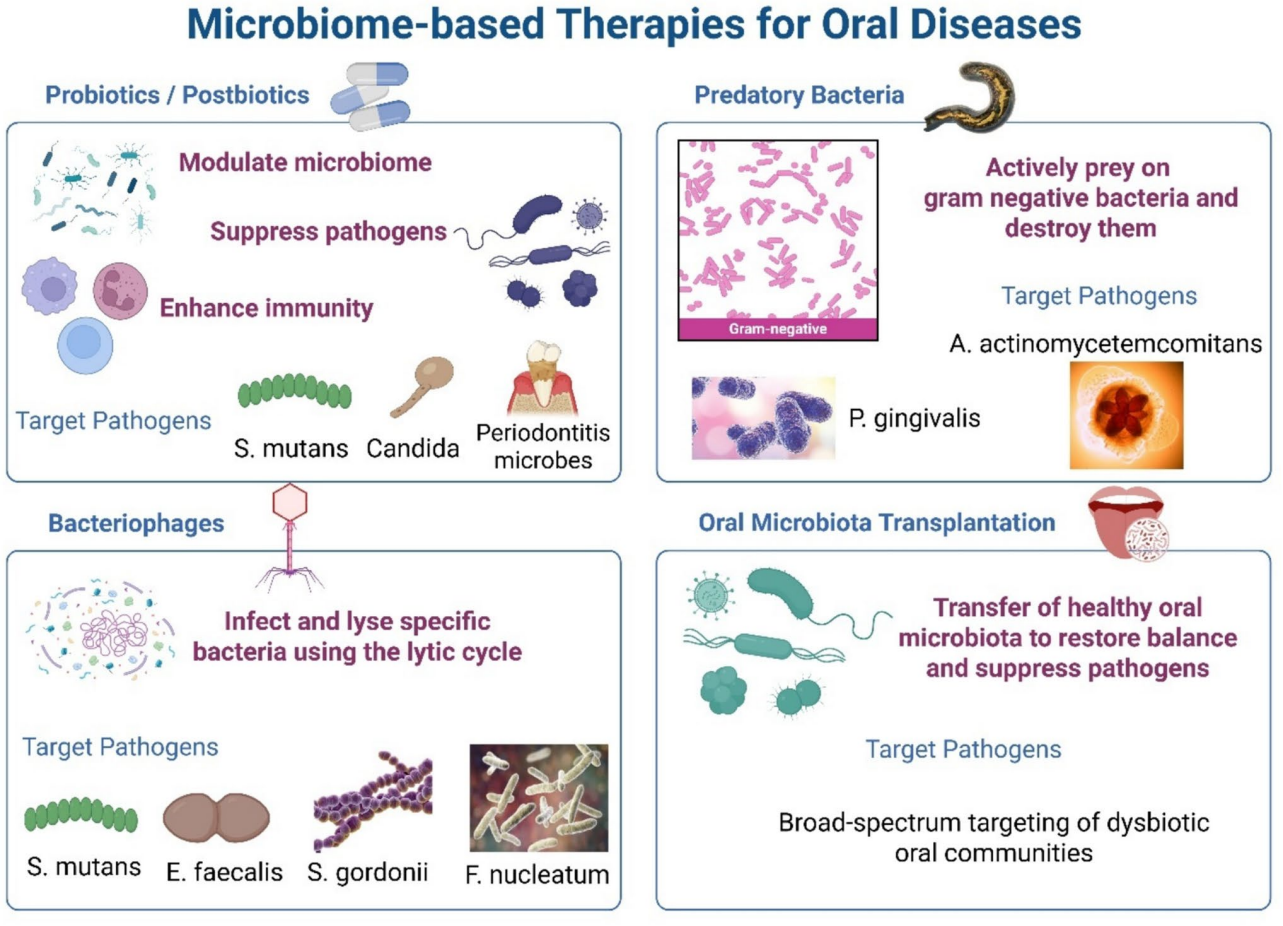
Harnessing the power of microbes to fight oral diseases—innovative therapies like probiotics, phages, and microbiota transplants reshape the mouth’s microbial battlefield

Probiotic therapy has attracted substantial interest due to its tremendous potential and optimistic prospects; however, it remains inadequately explored. Probiotics, as described by the Food and Agriculture Organization of the United Nations and the World Health Organization, are live microorganisms that, when administered in adequate amounts, confer a health benefit on the host (Tiwari et al. 2024). Over the past decade, multiple clinical investigations have examined the impact of probiotics on enhancing oral health. These studies encompass various applications, including the treatment of gingivitis, periodontitis, dental cavities, halitosis, and microbial equilibrium (Homayouni Rad et al. 2023). In 2019, the International Scientific Association for Probiotics and Prebiotics (ISAPP) defined “postbiotics” as a preparation of inanimate bacteria and/or their components that provides a health benefit to the host. Postbiotics are regarded as metabolites released by probiotic strains during fermentation, comprising microbial cell fractions, polypeptides, muropeptides, bacteriocins, peptidoglycan-derived peroxides, pili-type structures, short-chain fatty acids, teichoic acid, folate, vitamins, lactic acid, and extracellular polysaccharides (Salminen et al. 2021). This section summarizes some of the clinical research conducted in the last 5 years regarding the therapeutic potential of probiotics and postbiotics in improving oral health (Fig. 4).

Numerous studies have shown that probiotics can markedly diminish inflammation and enhance clinical indices in the management of gingivitis and periodontitis. In a double-blind controlled study conducted on school children aged 5–12 years and the elderly over 60 years, probiotic and xylitol mouth rinses decreased *Streptococcus mutans* (*S. mutans*) levels in both populations, with probiotics demonstrating greater efficacy in children (Krupa et al. 2022). Similarly, it was demonstrated that probiotic mouthwash was almost as effective as chlorhexidine in diminishing plaque and bleeding scores in chronic gingivitis patients (Boyapati et al. 2024). Two studies conducted by Babina and colleagues revealed that the use of *Streptococcus salivarius* M18 and K12 strains in young adults with gingivitis aided in diminishing gingival and plaque indices and in minimizing plaque accumulation, respectively (Babina et al. 2022, 2023). Likewise, a marked reduction in gingival hemorrhage among orthodontic patients using probiotic toothpaste was observed by others (Tahir et al. 2025). Several studies corroborated the efficacy of probiotics in conjunction with scale and root planning, revealing diverse levels of clinical and immunological advantages, such as reduced bleeding on probing, enhanced gingival indices and probing depths, reduced inflammatory markers including interleukins IL-1β and IL-8, an increase in IL-10 and TNF-α, elevated salivary IgA, and enhanced plaque management (Minić et al. 2022; Ramos et al. 2022; Ranjith et al. 2022; Salinas-Azuceno et al. 2022; Poulose et al. 2024). A significant immunological modulation by the probiotics *Bifidobacterium lactis* HN019 and *Bifidobacterium lactis* DN-173010, through enhanced expression of β-defensins and cytokine regulation, was also reported (Invernici et al. 2020; de Almeida Silva Levi et al. 2023; Özener et al. 2023). It was observed that although gingival bleeding was ameliorated in patients with periodontitis, deeper pockets remained with *Lactobacillus brevis* and *Lactobacillus plantarum* use (Pudgar et al. 2021). The use of the probiotic VSL#3 was evaluated in patients with oral lichen planus, a common chronic inflammatory disease of the oral mucosa, where a trend of reduced IFN-γ levels without significant clinical enhancement was observed (Marlina et al. 2022). Diminished bleeding scores in peri-implant mucositis were recorded following probiotic administration in another study (Sargolzaei et al. 2022).

Probiotics were found to have a beneficial effect in dental caries prevention. For example, the prolonged consumption of probiotic lozenges containing arginine decreased the risk of caries in youngsters (Pørksen et al. 2023). In children with severe early childhood caries (ECC), the use of *Lactobacillus paracasei* SD1 elevated antimicrobial peptide concentrations and reduced *S. mutans* (Wattanarat et al. 2021). A study conducted on the probiotic milk Yakult and the probiotic powder enKorD revealed that both products can prevent dental caries by lowering the oral microbial load of *S. mutans*, although the long-term effect and risks were still to be evaluated (Janiani and Ravindran 2022). The cariostatic effect of probiotic milk was also investigated in another study (Sandoval et al. 2021), where it decreased the prevalence of caries and lowered salivary β-defensin-3 levels. Additionally, probiotic tablets and solutions were found to significantly reduce early carious lesions and *S. mutans* levels, even after brief periods of administration (Patil et al. 2021; Olczak-Kowalczyk et al. 2025). Upon investigating the influence of *Lactiplantibacillus plantarum* CCFM8724 on the oral microbiota of children with dental caries, it was found that both *S. mutans* and *Candida albicans* were reduced, while the oral microbiome was positively altered (Zhang et al. 2023). A substantial reduction of *S. mutans* following 3 weeks of probiotic curd eating was previously observed in another study (Sakhare et al. 2021). Interestingly, *Streptococcus dentisani* 7746, isolated from dental plaque of caries-free individuals, showed antimicrobial activity against oral pathogens, decreasing plaque and gingival inflammation while enhancing good bacterial indicators and overall oral health (Ferrer et al. 2020).

Halitosis denotes unpleasant oral odor during exhalation, originating from the mouth, nasal cavity, upper respiratory tract, and upper digestive system. *Lactobacillus salivarius* G60, with or without inulin, demonstrated beneficial effects in mitigating halitosis and tongue coating (Mousquer et al. 2020). Similarly, *Weissella cibaria* CMU lozenges markedly reduced halitosis and improved oral health–related quality of life (Lee et al. 2021). This was also emphasized in a case study that reported the home use of oral products containing probiotics and paraprobiotics in maintaining tongue eubiosis, where halitosis was effectively treated (Cannizzaro et al. 2023).

Numerous studies utilized sequencing to evaluate microbiota regulation. Lozenges comprising *Lactobacillus salivarius*, *Lactobacillus paracasei*, and *Lactobacillus plantarum* enhanced beneficial bacteria, which ameliorated plaque and immunological indicators (Lin et al. 2022). The consumption of the probiotic-rich beverage “koumiss” increased oral microbial diversity while reducing Gram-negative and pathogenic microorganisms (Chen et al. 2024). Since xerostomia has a detrimental effect on oral microbiota of cancer patients undergoing radiotherapy, the efficacy of probiotics in maintaining oral health in cancer patients has been investigated. A study conducted on head and neck cancer patients has found slight, though not significant, alterations in microbiota upon the use of oral lozenges containing *Streptococcus salivarius*, along with an improvement in plaque index scores and periodontal screening. However, further study was recommended for establishing this data (Vesty et al. 2020).

Alongside live probiotics, some studies examined postbiotics in improving oral health. One study assessed the use of lozenges containing heat-killed strains of *L. salivarius*, *L. paracasei*, and *L. plantarum*, observing elevated *Lactobacillus* levels, enhanced salivary IgA, diminished plaque accumulation, and ameliorated subjective oral and gastrointestinal health symptoms (Lin et al. 2022). Additionally, the evaluation of both live and heat-inactivated *L. paracasei* (ET-22 strain) revealed that both variants markedly diminished halitosis-related bacteria such as *Rothia*, *Solobacterium*, and *Peptostreptococcus*, as well as the generation of volatile sulfur compounds (Wuri et al. 2023). These findings underscore that postbiotics—particularly heat-killed formulations—can be equally efficacious as live probiotics in altering oral microbiota and enhancing clinical outcomes, with additional benefits of safety and stability.

### Predatory bacteria

Using predatory bacteria, specifically *Bdellovibrio bacteriovorus* (BALOs), shows significant promise as a novel treatment strategy for oral diseases like periodontitis (Table 1). They can selectively target key pathogens such as gram-negative bacteria, disrupt biofilms, and modulate the host immune response. The oral environment, with its constant presence of saliva and largely anaerobic conditions, is not ideal for the survival or activity of these predators. However, research over the past decade has demonstrated that under certain conditions, BALOs can effectively reduce pathogenic bacteria associated with oral diseases (Van Essche et al. 2011) (Fig. 4).

**Table 1:** Examples of BALOs and their use to control oral pathogens

BALO	Oral pathogens	Effect	References
*B. bacteriovorus* 109J	*A. actinomycetemcomitans*,* E. corrodens*	Significantly reduce the planktonic growth and effectively eliminate their biofilms	(Dashiff and Kadouri 2011)
*B. bacteriovorus* HD100	*A. actinomycetemcomitans*, *F. nucleatum*	Infiltrating biofilms and decreasing bacterial populations in both free-floating and attached states	(Van Essche et al. 2009; Loozen et al. 2015)
*B. bacteriovorus* HD100	*E. coli E. corrodens*,* A. actinomycetemcomitans*	Targeting and consuming both aerobic and microaerophilic bacterial species	(Patini et al. 2019)
*B. bacteriovorus* HD100	Bacteria, e.g., *F. nucleatum*,* C. gracilis* in rat models of experimental periodontitis	Reduced periodontitis-related bacterial populations, minimized bone loss, enhanced bone structure, protected periodontal tissues, and modulated the immune response	(Silva et al. 2023)

#### Mechanisms of action

Upon contact with susceptible prey, *B. bacteriovorus* can either invade the periplasmic space and replicate intracellularly (in the case of periplasmic predators) or attach externally and degrade the host bacterium from the outside (as seen with epibiotic predators like *Micavibrio aeruginosavorus*). This direct predation leads to the destruction of the prey cell, effectively reducing the pathogenic bacterial load in the environment (Dashiff and Kadouri 2011). Importantly, BALOs are highly selective for Gram-negative bacteria and have no cytotoxic effects on eukaryotic cells. This selectivity ensures that host tissues remain unharmed and that beneficial Gram-positive commensals—crucial for maintaining oral microbial balance—are preserved. Moreover, these predatory organisms are self-limiting in nature. Once prey bacteria are depleted, the predators are unable to replicate, and their population declines naturally. This biological check reduces the risk of overgrowth and minimizes the chance of resistance development, which is a major concern with conventional antibiotics (Van Essche et al. 2011; Sinha et al. 2014).

#### Oral applications of predatory bacteria

Predatory bacteria offer promising therapeutic potential, particularly in combating periodontal diseases that are driven by Gram-negative anaerobes such as *Porphyromonas gingivalis* and *Aggregatibacter actinomycetemcomitans*. These pathogens are strongly implicated in the initiation and progression of periodontitis, where they contribute to inflammation, tissue destruction, and alveolar bone loss. Laboratory studies have demonstrated that *B. bacteriovorus* can effectively reduce the abundance of these bacteria in both planktonic and biofilm states, although complete eradication is often limited by environmental factors such as oxygen availability (Van Essche et al. 2009; Negus et al. 2017).

Delivery systems for these therapeutic agents are still under development and include formulations such as mouth rinses, gels, or slow-release devices applied locally to the gingival sulcus or periodontal pockets. These localized applications aim to maximize predator–pathogen interactions while minimizing systemic exposure. The use of BALOs in conjunction with other microbiome-modulating therapies, such as probiotics and prebiotics, may further enhance treatment outcomes. This synergistic approach could help restore a healthy microbial balance in the oral cavity, supporting both infection control and tissue healing through ecological rebalancing (Shatzkes et al. 2017).

### Bacteriophage

Bacteriophages offer another precision tool for targeting oral pathogens (Table 2 and Fig. 4). Phages can lyse bacteria upon replication (lytic cycle) or integrate into host genomes (lysogenic cycle). Lytic phages are preferred for therapeutic use (Shlezinger et al. 2017).

**Table 2:** Phage therapy in oral disease

Phages	Phage source	Target pathogen	Phage type	Key findings	References
I. Phage therapy against dental caries					
SMHBZ8	Saliva, teeth, dental plaque, dental sewage	*S. mutans*	Virulent (lytic)	– Strong lytic activity against *S. mutans* in dentin caries model	(Ben-Zaken et al. 2021)
SMHBZ8	Saliva, teeth, dental plaque, dental sewage		Virulent (lytic)	– Reduces *S. mutans* load and caries development	(Wolfoviz-Zilberman et al. 2021)
ɸAPCM01	Saliva		Virulent (lytic)	– Reduces *S. mutans* growth and biofilm formation	(Dalmasso et al. 2015)
II. Phage therapy against periapical periodontitis					
PEf771	Unsterilized wastewater	*E. faecalis*	Virulent (lytic)	– It is the first Myoviridae phage for *E. faecalis*, closely related to EFDG1; it uses a holin-endolysin lysis strategy	(Xiang et al. 2020)
PEf771	Sewage water		Virulent (lytic)	– Effectively prevents *E. faecalis* YN771 and RYN771 infections and shows superior antibacterial activity over antibiotics	(Xiang et al. 2022)
HEf13	Sewage water		Virulent (lytic)	– Strong lytic activity in an ex vivo dentin model	(Lee et al. 2019)
A novel phage of the Siphoviridae family	Sewage water		Virulent (lytic)	– Strong tool for treating *E. faecalis* in chronic periodontitis	(Bhardwaj et al. 2020)
vB_EfaS-SRH2	Wastewater samples		Virulent (lytic)	– Potential for treating odontogenic infections and resistant apical periodontitis	(Pazhouhnia et al. 2022)
III. Phage therapy against periodontal disease					
FNU1	Mouthwash water	*F. nucleatum*	Virulent (lytic)	– Effectively reduces biofilms and the potential for treating infections	
ΦSG005	Drainage water	*S. gordonii*	Virulent (lytic)	– Strong bactericidal activity against *S. gordonii*	

Some studies have explored the clinical potential of bacteriophages targeting *Streptococcus mutans*, a key cariogenic pathogen. Two distinct lytic phages, SMHBZ8 and ɸAPCM01, were isolated from diverse oral and environmental samples and demonstrated strong antimicrobial activity against *S. mutans*, particularly type c strains. SMHBZ8 effectively reduced bacterial viability and inhibited biofilm formation at a high MOI, while in vitro and murine in vivo experiments confirmed its ability to prevent caries development without toxicity to mouse macrophages. Similarly, ɸAPCM01, isolated from saliva, showed a burst size of approximately 44 phages per infected cell and significantly reduced the metabolic activity of *S. mutans* biofilms (Dalmasso et al. 2015; Ben-Zaken et al. 2021; Wolfoviz-Zilberman et al. 2021).

Several studies from China and other regions have demonstrated the strong therapeutic potential of various bacteriophages against *Enterococcus faecalis*, a key pathogen in endodontic and oral infections. Phage PEf771, characterized by a short latent period and burst size of 78, showed superior antibacterial efficacy compared to 10 antibiotics and 3 disinfectants in both in vitro root canal and in vivo rat models, without harming vital organs. Similarly, phages vB_EfaS-SRH2 and HEf13 exhibited short latent phases, large burst sizes (125 and 352, respectively), and high lytic activity against *E. faecalis*, including biofilm-forming, drug-resistant strains. Notably, phage HEf13 effectively reduced bacterial load in an ex vivo dentin model and lacked harmful genes, indicating good safety potential. Despite these promising results, only a few studies reported on safety, with most omitting data on adverse effects (Lee et al. 2019; Xiang et al. 2020, 2022).

Research from Australia and Japan each targeted different oral pathogens: *Fusobacterium nucleatum* and *Streptococcus gordonii*, respectively. In the Japanese study, researchers discovered a new virulent phage, ΦSG005, which specifically infects *S. gordonii*. The phage has a 16,127-bp DNA genome with 21 predicted genes and forms a unique clade among known streptococcal phages. Notably, it encodes an AcrIIA5-like protein that can inhibit type II Cas9, acting as a defense mechanism against bacterial immune systems. Meanwhile, the Australian study demonstrated that phage FNU1 can effectively lyse *F. nucleatum* cells and break down their biofilms, indicating its therapeutic potential for treating periodontitis (Kabwe et al. 2019; Fujiki et al. 2021).

### Oral microbiota transplantation

Oral microbiota transplantation (OMT) is a promising new approach modeled after fecal microbiota transplantation (FMT), aimed at treating periodontal disease by restoring a balanced microbial community in the oral cavity. This technique involves transferring beneficial oral microorganisms from healthy individuals to those with periodontal disease to help reduce inflammation, protect periodontal tissues from further damage, and re-establish a healthy microbial environment. While successful results have been seen in dogs with naturally occurring periodontitis, OMT has not yet been applied in human clinical settings due to practical and safety considerations (Nascimento 2017; Goloshchapov et al. 2024; Lindo et al. 2024) (Fig. 4).

Further research by Pozhitkov et al. highlighted distinct microbial compositions in individuals with different oral health statuses. Healthy individuals showed dominance of *P. melaninogenica*, *F. nucleatum*, *Neisseria elongata*, and various *Capnocytophaga* species, while periodontitis patients had higher microbial diversity and were dominated by pathogens like *Prevotella intermedia*, *T. vincentii*, and uncultured *Porphyromonas* spp. These findings support the potential of OMT as a targeted, microbiome-based therapy for periodontal disease (Pozhitkov et al. 2015).

A pilot clinical study demonstrates that oral microbiota transplantation using maternal saliva is a safe and potentially effective approach for preventing severe chemotherapy-induced oral mucositis in pediatric patients with neuroblastoma. The procedure not only reduced the severity of mucositis during subsequent chemotherapy and post-transplant conditioning but also resulted in beneficial shifts in the oral microbial composition. The absence of adverse effects further supports the feasibility of this intervention, highlighting its potential as a supportive care strategy in oncology settings (Goloshchapov et al. 2024). Another study demonstrates the feasibility of oral microbiota transplantation (OMT) as a potential treatment for intra-oral halitosis (IOH). Transplantation of salivary flora from IOH patients into Wistar rats led to colonization by odor-associated bacteria, increased IOH-related metabolic activity, and elevated breath values. Conversely, transplantation of healthy oral microbiota resulted in reduced colonization by malodor-producing bacteria and significantly lower breath scores. These findings suggest that restoring oral microbial balance through OMT may be an effective strategy for mitigating IOH and warrant further investigation in clinical settings (Huang and Cheng 2024).

Although it is promising, there are still limitations for applying microbiome-based therapies; these limitations start from the microbiome resilience, including strain selection, donor screening as a host factor, ambiguous mechanisms of action, and the risk of adverse effects. The intricate nature of the human gut microbiome and its interactions with the host complicate the translation of research into successful treatment interventions (Hwang et al. 2017).

Microbiome-based therapies, particularly fecal microbiota transplantation (FMT), exhibit several limitations, including diversity in microbiota composition among individuals, the potential for transferring pathogenic microbes, and difficulties in standardizing collection, preparation, and storage protocols (Hwang et al. 2017). Predatory bacteria exhibit a restricted host range, vulnerability to host defenses and antibiotics, and the possibility of eliciting allergic reactions (Adams et al. 2017). Moreover, certain predatory bacteria are incapable of preying on Gram-positive bacteria, which significantly contribute to eye infections. Comprehending the mechanisms of predation and their effects on bacterial populations poses hurdles as well (Inchingolo et al. 2025). Furthermore, there exists a challenge in accurately describing and quantifying postbiotic and probiotic constituents, a deficiency in manufacturing standardization and safety assessments, and a necessity for enhanced comprehension of their methods of action and potential health effects (Azzam et al. 2020).

## Conclusion

The intricate interplay between the oral microbiome and host tissues is central to maintaining oral health, while dysbiotic shifts serve as key triggers for a variety of oral diseases. Traditional antimicrobial therapies, though effective, often lack specificity and can disrupt beneficial microbial communities. Microbiome-targeted approaches, including probiotics, postbiotics, and predatory bacteria, represent a paradigm shift toward ecological management of oral health, emphasizing the restoration and maintenance of microbial balance rather than mere pathogen eradication. Evidence highlights that probiotic strains such as *Streptococcus salivarius* and *Lactobacillus* spp., whether live or heat inactivated, can reduce pathogenic bacteria and inflammation and improve clinical indicators in conditions like gingivitis and periodontitis. Similarly, predatory bacteria like *Bdellovibrio bacteriovorus* offer a novel, targeted method for controlling biofilm-forming pathogens, especially Gram-negative species. Despite these advancements, challenges remain, including the need for standardized protocols, precise dosing, understanding strain-specific effects, and evaluating long-term safety profiles. Future research should focus on personalized microbiome modulation strategies, integrating host genetics, microbiota composition, and environmental factors to develop tailored treatments, ultimately advancing holistic oral healthcare.

## Future research directions

Future research should focus on elucidating the complex interactions within the oral microbiome, identifying keystone microbial species, and understanding their influence on systemic health. The development of precision microbiota-based therapeutics tailored to individual microbial profiles has great potential. Additionally, exploring innovative delivery systems for probiotics, postbiotics, and predatory bacteria could enhance efficacy and stability. Large-scale, well-designed clinical trials are essential to validate these approaches and facilitate their integration into routine dental practice. Ultimately, advancing our understanding and manipulation of the oral microbiome offers a promising pathway toward improved oral and overall health outcomes.

## Data Availability

Data sharing is not applicable to this article as no new data were created or analyzed in this study.

## Abbreviations

AhR: Aryl hydrocarbon receptor
ALS: Agglutinin-like sequence
BALOs: *Bdellovibrio bacteriovorus*
FISH: Fluorescence in situ hybridization
FMT: Fecal microbiota transplantation
H₂S: Hydrogen sulfide
HWP: Hyphal wall protein
ISAPP: International Scientific Association for Probiotics and Prebiotics
LPS: Lipopolysaccharide
MRONJ: Medication-related osteonecrosis of the jaw
OMT: Oral microbiota transplantation
SAPs: Secreted aspartyl proteinases
SCFAs: Short-chain fatty acids
TMJ: Temporomandibular joint
TRFLP: Terminal-restriction fragment length polymorphism
VSCs: Volatile sulfur compounds
